# RNAi-Mediated Suppression of *OsBBTI5* Promotes Salt Stress Tolerance in Rice

**DOI:** 10.3390/ijms25021284

**Published:** 2024-01-20

**Authors:** Zhimin Lin, Xiaoyan Yi, Muhammad Moaaz Ali, Lijuan Zhang, Shaojuan Wang, Shengnan Tian, Faxing Chen

**Affiliations:** 1 Fujian Academy of Agricultural Sciences Biotechnology Institute, Fuzhou 350003, China; 2 College of Horticulture, Fujian Agriculture and Forestry University, Fuzhou 350002, China; 1210305019@fafu.edu.cn (X.Y.); moaaz@fafu.edu.cn (M.M.A.); 1210305020@fafu.edu.cn (L.Z.); 3210330057@fafu.edu.cn (S.W.); 3210330052@fafu.edu.cn (S.T.)

**Keywords:** *OsBBTI5*, rice, 24-epibrassinolide, salt tolerance, RNAi, transcriptome

## Abstract

This study explores the impact of RNAi in terms of selectively inhibiting the expression of the *OsBBTI5* gene, with the primary objective of uncovering its involvement in the molecular mechanisms associated with salt tolerance in rice. *OsBBTI5*, belonging to the Bowman–Birk inhibitor (BBI) family gene, is known for its involvement in plant stress responses. The gene was successfully cloned from rice, exhibiting transcriptional self-activation in yeast. A yeast two-hybrid assay confirmed its specific binding to *OsAPX2* (an ascorbate peroxidase gene). Transgenic *OsBBTI5*-RNAi plants displayed insensitivity to varying concentrations of 24-epibrassinolide in the brassinosteroid sensitivity assay. However, they showed reduced root and plant height at high concentrations (10 and 100 µM) of GA_3_ immersion. Enzyme activity assays revealed increased peroxidase (POD) and superoxide dismutase (SOD) activities and decreased malondialdehyde (MDA) content under 40-60 mM NaCl. Transcriptomic analysis indicated a significant upregulation of photosynthesis-related genes in transgenic plants under salt stress compared to the wild type. Notably, this study provides novel insights, suggesting that the BBI gene is part of the BR signaling pathway, and that *OsBBTI5* potentially enhances stress tolerance in transgenic plants through interaction with the salt stress-related gene *OsAPX2*.

## 1. Introduction

Abiotic stresses typically exert a direct influence on various facets of plant growth, developmental processes, and ultimate crop yield outcomes [1,2]. Salinity stress is one of the major abiotic stresses [3]. Elevated salinity primarily triggers ionic and osmotic stress in plants, adversely affecting plant cells by disrupting crucial cellular processes such as photosynthesis and promoting the generation of reactive oxygen species (ROS) [4,5]. In the natural environment, dicotyledons exhibit a more extensive range of variation in salinity tolerance compared to monocotyledons. Within the plant kingdom, barley stands out as the most salinity-tolerant species, while wheat typically displays a moderate level of tolerance. Among cereals, rice is notably the most susceptible to salinity stress [6]. Moreover, it is noteworthy that the yield of rice is considerably more affected by salt stress than by the overall growth of the plant [7]. Consequently, there has been a considerable focus on researching salt tolerance in rice, as evidenced by numerous studies [8,9,10]. Currently, advances in whole genome sequencing, marker-assisted breeding strategies and targeted mutagenesis have greatly improved the tools available for rice breeding [11,12,13,14,15]. Using the whole-genome sequencing analysis of two salt-tolerant (Pokkali and Nona-Bokra) and three salt-sensitive rice varieties (Bengal, Cocodrie and IR64), and employing a combination of quantitative trait locus (QTL) localization and expression profiling data, a total of 396 differentially expressed genes were identified within the coding region [16]. 

The B-type response regulator *hst1* (hitomebore salt-tolerant 1) controls salinity tolerance in rice by regulating transcription factors and antioxidant mechanisms [17]. The introduction of the *hst1* gene into rice, coupled with the application of single-nucleotide polymorphism (SNP) marker-assisted selection, led to the development of the BC3FC population YNU31-24, which exhibited a genomic similarity of 93.5% to the parental varieties. YNU31-24 seedlings demonstrated enhanced survival and increased plant biomass when exposed to 125 mM NaCl [12]. A deletion mutant exclusively targeting the salt tolerance (*DST* genes) was generated in the indica rice variety MTU1010 through CRISPR-Cas9 gene editing. This modification led to observable phenotypic changes, including broader leaves, diminished stomatal density, heightened leaf water retention, and a discernible tolerance to osmotic stress and high salt stress during the seedling stage of the mutant [18]. In addition, T-DNA insertion mutagenesis and RNAi silencing of target genes are also methods used to improve salt tolerance in rice [19,20].

One of the many important physiological changes during the early evolution of plant cells was the ability to adapt to low levels of Na^+^ and K^+^ intermediates [21]. Cellular Na^+^ toxicity is the ionic toxicity that primarily causes salt stress and usually results in a variety of physiological processes in rice, including K^+^ attraction [22]. In rice, intracellular Na+ is mainly transported out of the cell via the SOS1 transporter protein or to the root xylem via the high-affinity potassium transporter proteins (HKT1;4, HKT1;5) to mitigate Na^+^ toxicity in the stem [23]. In rice, the role of HKT2;1 may be to provide an entry pathway for Na^+^ uptake under K^+^ limitations, mainly to support cell expansion and plant growth [24]. In plants, superior K^+^ retention in salt-stressed roots is positively correlated with salt tolerance [25]. In rice, the K^+^ transporter proteins OsHAK1 and OsHAK5, when induced by salt stress, mediate K^+^ uptake and transport, maintaining a high K^+^/Na^+^ ratio under salt stress [26,27]. Studies have shown that most transcription factors (TFs) are involved in the response to salt stress, such as *AP2/ERF*, *bHLH*, *NAC*, *MYB* and *bZIP* [28,29,30,31,32]. For example, overexpression of rice *AP2/ERF-type* TFs in *Arabidopsis thaliana* showed improved tolerance to drought, salt and cold [33]. In addition, many hormone-like genes in rice are involved in the salt stress response. Overexpression of the rice Aux/IAA TF *OsIAA17* significantly increases salt and drought tolerance in rice [34]. Knockdown of *OsABI5*, a gene downstream of the abscisic acid signaling pathway in rice, resulted in delayed seed germination and reduced seedling salt tolerance [35]. Overexpression of *OsGA2ox5* increased the resistance of rice to high-salinity stress [36]. 

Brassinolide (BR) is an asterol compound that, when soaked, inhibits the accumulation of ROS, reduces the level of MDA, increases the SPAD of rice seedlings under NaCl stress, and protects the plant’s photosynthetic system [37,38].

The Bowman–Birk Inhibitor (BBI) encodes a serine protease inhibitor and has a repetitive cysteine-rich structural domain with a reactive site from the trypsin or chymotrypsin family [39]. BBIs are generally well known in soybeans and their main function is reflected in their role in inhibiting the proliferation of cancer cells [40]. Moreover, BBIs in wheat and maize mainly show strong inhibitory effects on the growth of plant pathogenic fungi [41]. In rice, there are currently 12 *BBI* genes [42], and there is limited understanding of their functions. For instance, the overexpression of the BBI protein APIP4 in rice has been shown to enhance resistance against the fungal pathogen *Magnaporthe oryzae* [43]. The hypothesis of this study is that the newly identified salt-inducible BBI, *OsBBTI5*, plays a crucial role in regulating salt tolerance in rice seedlings. In the current study, *OsBBTI5* gene, when suppressed through RNAi in transgenic plants, produced a positive enhancement of salt tolerance. Our investigation also aimed to understand the molecular mechanisms underlying *OsBBTI5*’s involvement in salt tolerance, particularly its association with the brassinosteroid (BR) pathway. Despite exhibiting insensitivity to BR, *OsBBTI5* showed remarkable sensitivity to the downstream gibberellic acid (GA_3_), leading to the inhibition of root system growth at high concentrations of GA_3_. Furthermore, physical interaction of *OsBBTI5* with *OsAPX2* was crucial in mediating its effects on salt tolerance. *OsBBTI5* regulated ROS accumulation and GA_3_ synthesis under salt stress conditions, implying a dual role in oxidative stress management and hormonal signaling. Thus, lowering the expression of *OsBBTI5* enhanced salt tolerance in rice seedlings by modulating H_2_O_2_ and GA_3_ signaling pathways. The selection of *OsBBTI5* among other genes was based on its novel identification as a salt-inducible BBI and our preliminary findings suggesting its significant impact on salt tolerance through intricate interactions with hormonal pathways and ROS regulation.

## 2. Results

### 2.1. In Silico Analysis of Rice BBI Genes

**Figure 1:**
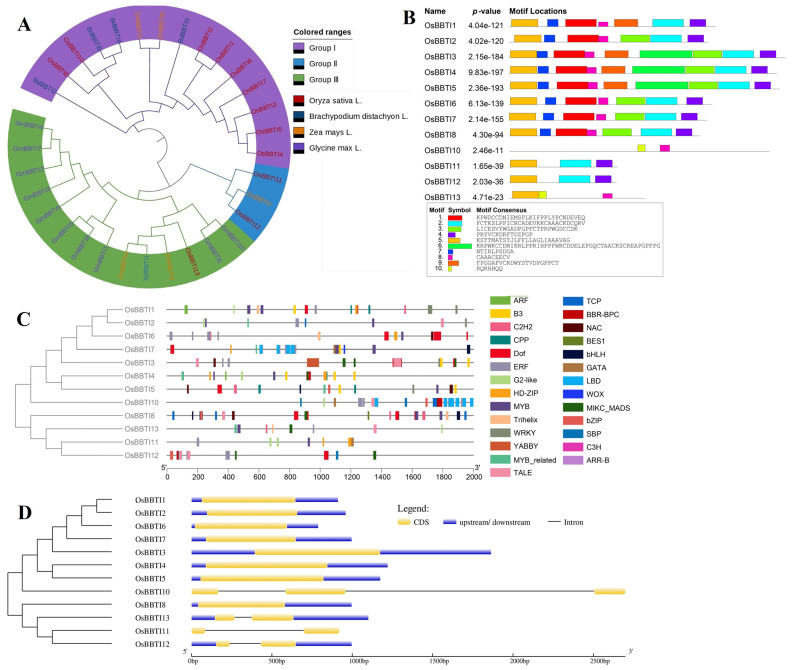
(**A**) Cluster analysis of the rice *BBI* family with homologs from *Brachypodium distachyon*, *Zea mays* and *Glycine max*. The four species are represented in red, blue, orange, and purple, respectively. (**B**) Structural domain analysis of BBI amino acids in rice. (**C**) Analysis of transcription factor binding sites of *BBI* family genes in rice. (**D**) Exon analysis of rice BBI family genes.

The full-length coding sequence (CDS) of *OsBBTI5* (Os01g0124401) was cloned based on Rice Genome Annotation Project (http://rice.uga.edu/index.shtml, accessed on 11 January 2023). Using the 12 BBI amino acid sequences of rice (*Oryza sativa* L.) as a query and searching for their homologues in phragmites (*Brachypodium distachyon* L.), maize (*Zea mays* L.) and soybean (*Glycine max* L.), we obtained 5, 5 and 10 homologues, respectively. These proteins, together with *OsBBTI5*, were used to construct the phylogenetic tree (Figure 1A). Phylogenetic analyses showed that *OsBBTI5* had the highest homology with the *OsBBTI4* gene in rice. It is closely related to *BdBBTI3* in *Brachypodium distachyon* L. and to *ZmBBTI5* and *ZmBBTI11* in maize. The *BBI* genes in rice are distributed between 2 and 9 motifs, with *OsBBTI5* having 9 motifs, according to structural domain analysis (Figure 1B). Analysis of the promoter regions of rice *BBI* genes showed that they have a total of 27 transcription factor binding sites, including the common C2H2, Dof, MYB, WRKY, NAC, BES1, bHLH, bZIP, and others (Figure 1C). Structural analysis of the rice *BBI* family genes showed that most of the *BBI* genes had only one exon, including *OsBBTI5*, with the exception of *OsBBTI11*, *OsBBTI12* and *OsBBTI13*, which had two exons, and *OsBBTI10*, which had three exons (Figure 1D).

### 2.2. Sub-Cellular Localization of OsBBTI5

**Figure 2:**
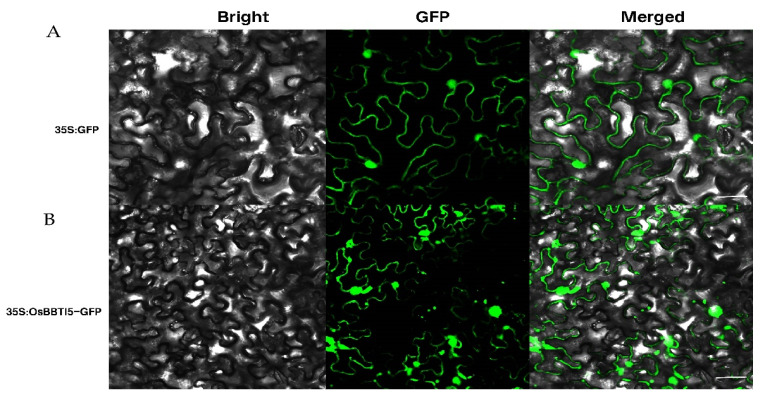
(**A**) Subcellular localization of the GFP protein in tobacco leaf epidermal cells, scale bar: 20 μm. (**B**) Subcellular localization of the OsBBTI5 protein in tobacco leaf epidermal cells, scale bar: 20 μm.

To determine the subcellular localization of *OsBBTI5* protein, the *OsBBTI5*-GFP recombinant plasmid of pCXSN was generated by fusing the *OsBBTI5* cDNA, lacking the termination codon, to the 5′ end of the GFP reporter gene under the control of the CaMV-35S promoter. The results showed that *OsBBTI5* expression in cells was distributed across organelles (Figure 2B), similar to the 35S:GFP (control) (Figure 2A). 

### 2.3. Characterization of OsBBTI5-RNAi Lines 

**Figure 3:**
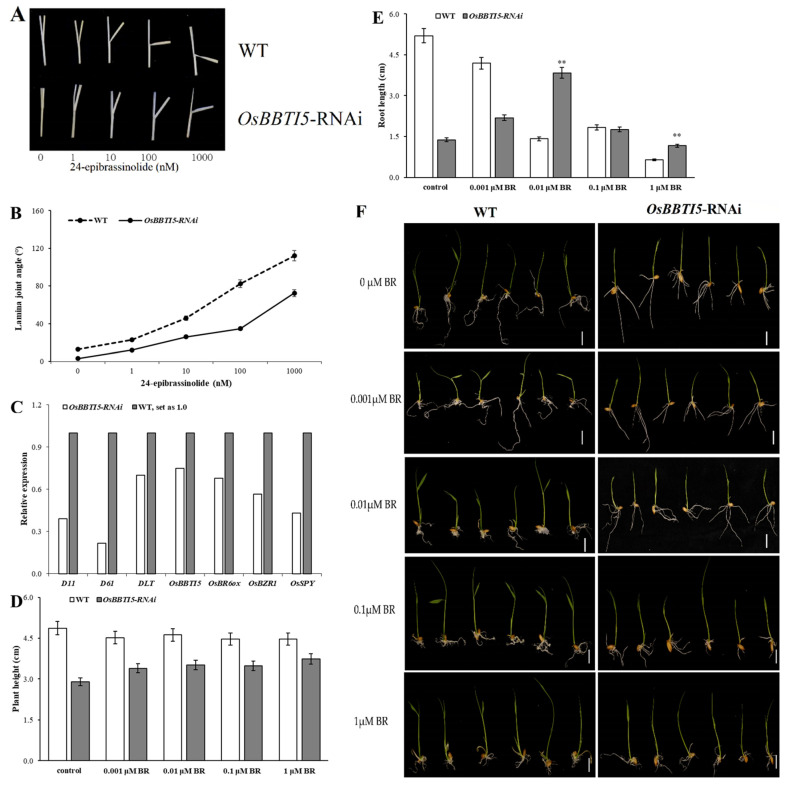
(**A**) The leaf inclination of *OsBBTI5*-RNAi and WT in the presence of indicated concentrations of 24-epibrassinolide. (**B**) Statistical analysis of leaf inclination, data are means ±SE (*n* = 20). (**C**) Comparison of BR-related gene expression between WT and *OsBBTI5*-RNAi. (**D**,**E**) Measurements of the plant heights and root lengths of WT and *OsBBTI5*-RNAi transgenic seedlings after 7 days of growing on an MS medium containing different 24-epibrassinolide concentrations. Data are means ± SDs (*n* = 30). (**F**) The growth of WT and *OsBBTI5*-RNAi plants grown on an MS medium containing different 24-epibrassinolide concentration after 7 days. ** *p* < 0.01. Scale = 1 cm.

To further validate the role of *OsBBTI5* in regulating BR signaling, we generated *OsBBTI5*-RNAi lines through RNAi knockdown technology. A BR sensitivity assay revealed that *OsBBTI5*-RNAi plants exhibited insensitivity to BR compared to the wild type (WT) (Figure 3A,B). Subsequently, seeds from both WT and transgenic plants were germinated and placed on a 0.5 × MS agar medium containing 0, 0.001 μM, 0.01 μM, 0.1 μM, or 1 μM 24-epibrassinolide.

The expression of BR biosynthesis-related genes, including *D11*, *D61*, *DLT*, *OsBBTI5*, *OsBR6ox*, *OsBZR1* and *OsSPY* genes, was analyzed via fluorescence quantitative PCR. The results showed different cumulative transcriptions of BR biosynthesis-related genes in the *OsBBTI5*-RNAi line (Figure 3C).

When cultivated on a medium containing 0 and 1 μM 24-epibrassinolide, no discernible differences in plant height or root length were observed between wild-type and transgenic *OsBBTI5*-RNAi plants (Figure 3D,F). However, in terms of root length, the transgenic *OsBBTI5*-RNAi showed significant differences compared to the wild type at 0.01 μM and 1 μM, indicating reduced sensitivity to 24-epibrassinolide (Figure 3E,F).

*OsBBTI5*-RNAi transgenic plants exhibited slower growth compared to the wild type at 0.1 μM, 1 μM, 10 μM, and 100 μM. Notably, transgenic plants were significantly shorter than the wild type in both plant height and root length when the medium contained 10 μM and 100 μM GA_3_ (Figure S1A,B). Overall, *OsBBTI5*-RNAi transgenic plants exhibited involvement in the BR pathway, displaying insensitivity to BR and sensitivity to high concentrations of GA_3_, with a predominant phenotype characterized by the absence of root growth (Figure S1C). 

### 2.4. Effect of NaCl Stress on Seed Germination

**Figure 4:**
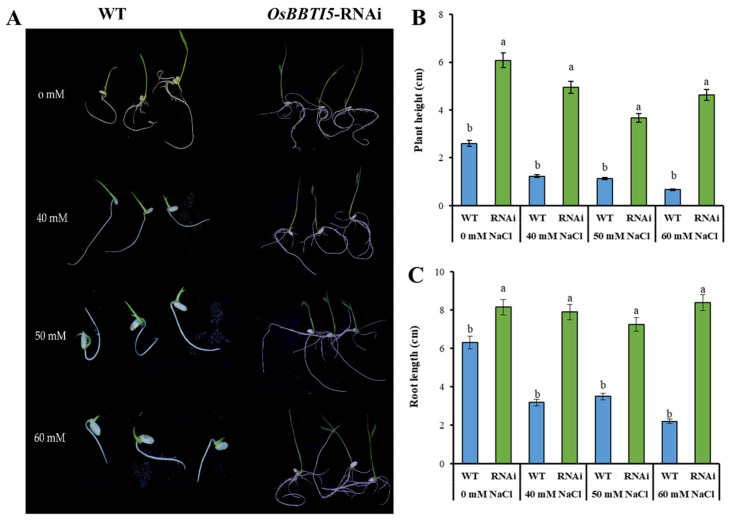
(**A**) Photographs of WT and *OsBBTI5*-RNAi transgenic seedlings supplemented with 0 mM, 40 mM, 50 mM, or 60 mM NaCl after 7 days of initiation. (**B**) Differences in plant height were compared between WT and *OsBBTI5*-RNAi transgenic plants. (**C**) Differences in root length were compared between wild-type and RNAi transgenic plants. Data represent means ± SD (*n* = 3). Different letters indicate significant differences (Tukey’s HSD test, *p* ≤ 0.05).

To determine salt stress in *OsBBTI5*-RNAi plants, seeds of WT and transgenic plants were germinated and then placed on a half-strength Murashige and Skoog (MS) medium containing 0 mM, 40 mM, 50 mM, or 60 mM NaCl. Under normal conditions, the transgenic seeds germinated faster and had greater plant height and root length than the wild type. The transgenic plants showed greater plant height and root length than WT when grown in different concentrations of salt solution (Figure 4A,B,C).

### 2.5. OsBBTI5-RNAi Enhances Salt Tolerance in Transgenic Rice

**Figure 5:**
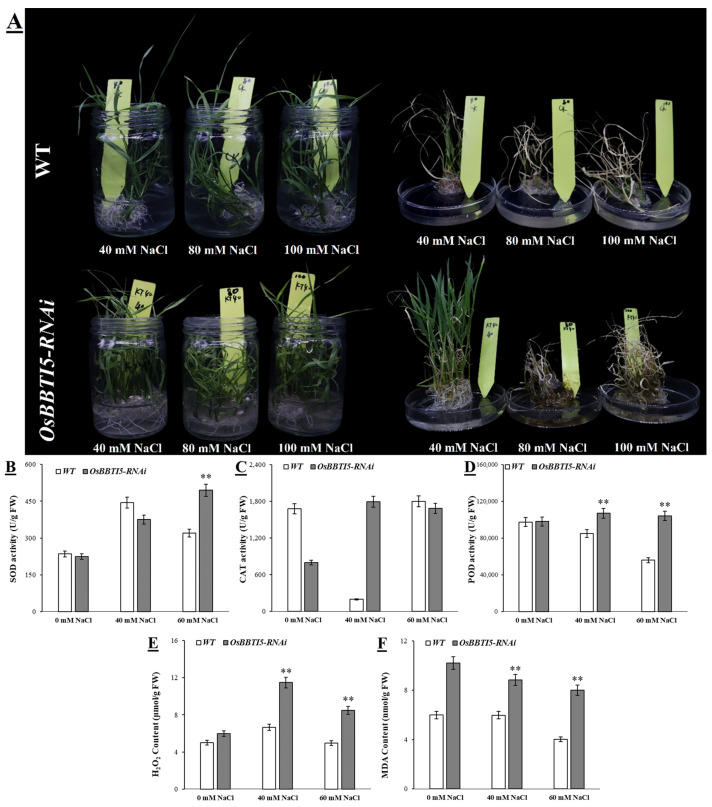
(**A**) Phenotypes of WT and transgenic plants in response to salt stress. (**B**) SOD activity. (**C**) CAT content. (**D**) POD content. (**E**) H_2_O_2_ content. (**F**) MDA content. Three independent experiments were carried out with similar results. Data represent means ± SD (*n* = 30). ** *p* ≤ 0.01.

*OsBBTI5*-RNAi plants were exposed to salt stress with different concentrations of NaCl for 7 days. As shown in Figure 5A, transgenic plants showed strong tolerance compared to WT after exposure to salt stress at a concentration of 40 mM. Under normal conditions without salt treatment, peroxidase (POD) and superoxide dismutase (SOD) activities were almost the same in *OsBBTI5*-RNAi and WT plants. Following salt stress treatment, *OsBBTI5*-RNAi plants exposed to 60 mM NaCl exhibited significantly higher activities of peroxidase (POD) and superoxide dismutase (SOD) compared to wild-type plants. However, at 40 mM NaCl, only the activity of POD was higher in *OsBBTI5*-RNAi plants than in the wild-type plants (Figure 5B,D). The activity of another antioxidant enzyme, CAT, was significantly increased in all seedlings at 40 mM NaCl after 7 days of salt treatment compared to control conditions, while the difference was not significant at 60 mM NaCl (Figure 5C). H_2_O_2_ was significantly higher in both 40 mM NaCl and 60 mM NaCl, whereas it was essentially indistinguishable in the no-salt condition (Figure 5E). Moreover, under normal conditions, the malondialdehyde (MDA) level, serving as an indicator of lipid peroxidation, was higher in *OsBBTI5*-RNAi compared to WT. Furthermore, the MDA content in *OsBBTI5*-RNAi plants was significantly elevated compared to wild-type plants, signifying an increased level of lipid peroxidation in transgenic plants under both 40 mM NaCl and 60 mM NaCl salt stress conditions (Figure 5F).

### 2.6. Differentially Expressed Genes Regulated by OsBBTI5 in Response to Salt Stress

RNA sequencing primarily involved three treatment groups: the wild-type (WT) group, the wild-type post-40 mM NaCl treatment (CK4), and *OsBBTI5*-RNAi after-40 mM NaCl treatment (KT39). Each sample yielded sequences ranging from 6.02 to 8.66 Gb, which underwent quality control using FASTQC. The results indicated that 92.12% to 95.12% of the sequences exhibited quality scores above Q30, and 62.69% to 95.05% of the reads were uniquely aligned with the genome (Supplementary Table S1). The alignment ratios were found to be similar across the three lines. Consequently, the analysis of differentially expressed genes (DEGs) was carried out using this consistent alignment ratio for further investigation. 

**Figure 6:**
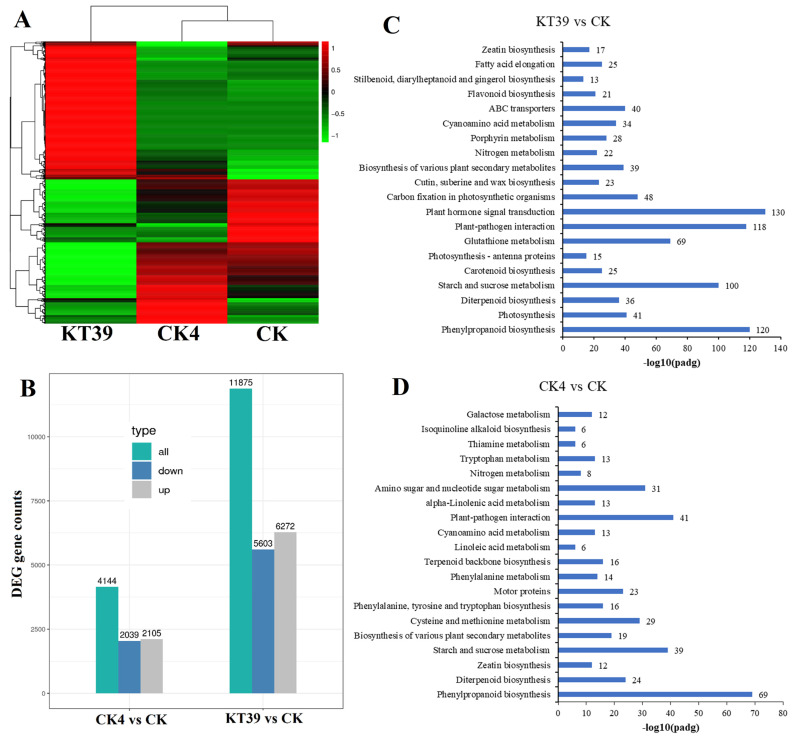
(**A**) Heat map of DEGs’ expressions in response to salt stress in two salt-stressed and WT samples. (**B**) Upregulated and downregulated DEGs comparison between two groups. (**C**) KEGG enrichment analysis between KT39 and CK group. (**D**) KEGG enrichment analysis between CK4 and CK group. KT39 is a synonym with the *OsBBTI5*-RNAi lines under 40 mM NaCl treatment; CK4 is a synonym with the wild-type lines under 40 mM NaCl treatment; and CK is a synonym with the wild-type lines under treatment with a normal nutrient solution.

DEGs were assessed by comparing the number of reads between the control and salt-treated samples in each line. Heat map analysis revealed significant differences in gene expression between the three lines (Figure 6A). Under 40 mM NaCl treatment, the expression level of *OsBBTI5*-RNAi was twofold higher compared to the salt-stressed WT, as observed in the analysis of DEGs in the WT (Figure 6B). Between salt-stressed WT and non-stressed WT, 2105 genes were upregulated and 2039 genes were downregulated. In contrast, between salt-stressed *OsBBTI5*-RNAi and WT, 5603 genes were upregulated and 6272 genes were downregulated. KEGG pathway enrichment analysis revealed that the phenylpropanoid biosynthesis pathway was best to be regulated under salt stress in rice (Figure 6C,D). Certainly, the most differential pathways between salt-stressed *OsBBTI5*-RNAi and salt-stressed WT were mainly focused on light-related pathways, including photosynthesis and photosynthesis-antenna proteins (Supplementary Tables S2 and S3). In addition, photosynthesis-related genes in particular showed significant upregulated expression (Supplementary Figures S2 and S3). GO enrichment analysis also revealed drastic changes in light-related pathways (Supplementary Figure S4), including photosynthesis, photosystem, and photosystem II. These findings support the hypothesis that distinct pathways are either activated or repressed in response to salt stress in *OsBBTI5*-RNAi compared to the wild type of rice.

### 2.7. In Vitro Interaction of OsBBTI5 with OsAPX2

**Figure 7:**
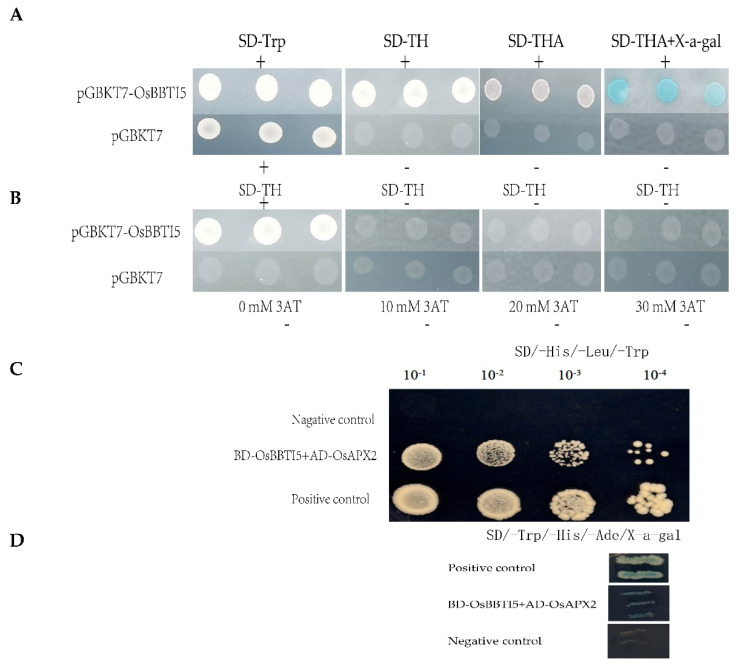
(**A**) Self-activating of *OsBBTI5*. +:pGBKT7-*OsBBTI5* (positive control), −:pGBKT7 (negative control). (**B**) Inhibitory effect of 3-AT. (**C**) Yeast two-hybrid assays for the interaction between *OsBBTI5* and *OsAPX2*. These strains were grown on SD-His-Leu-Trp. (**D**) Yeast two-hybrid assays for the interaction between *OsBBTI5* and *OsAPX2*. These strains were grown on SD-His-Leu-Trp + X-α-Gal.

In order to understand whether the *OsBBTI5* gene was self-activating, we carried out its self-activation experiments. On SD/-Trp, SD/-Trp/-His, SD/-Trp/-His/-Ade, and SD/-Trp/-His/-Ade+X-α-gal plates, pGBKT7-B5 was able to grow, whereas pGBKT7-negative was not able to grow, suggesting that there is self-activation of pGBKT7-B5 (Figure 7A). Inhibition experiments of 3-AT showed that the self-activation of pGBKT7-*OsBBTI5* could be effectively inhibited in 3-AT-deficient plates at concentrations higher than 10 mM (Figure 7B). We previously performed an interaction screening of *OsBBTI5* proteins against the rice yeast library and obtained 96 candidate proteins, including *OsAPX2*. To elucidate the molecular mechanism underlying salt tolerance in rice seedlings mediated by *OsBBTI5*, we conducted yeast two-hybrid library screening, using *OsBBTI5* as bait. *OsAPX2*, an ascorbate peroxidase gene in rice, was identified as an *OsBBTI5*-interacting protein by an *OsBBTI5* bait protein screen (Figure 7C,D).

## 3. Discussion

The majority of structures within the rice *BBI* genes exhibit conserved coding sequences (CDS) without introns (Figure 1). These inhibitor families operate through specific mechanisms of enzyme hydrolysis and are categorized based on the active amino acid in their reaction center, such as serine, cysteine, aspartic, and metalloproteases [44].

**Figure 8:**
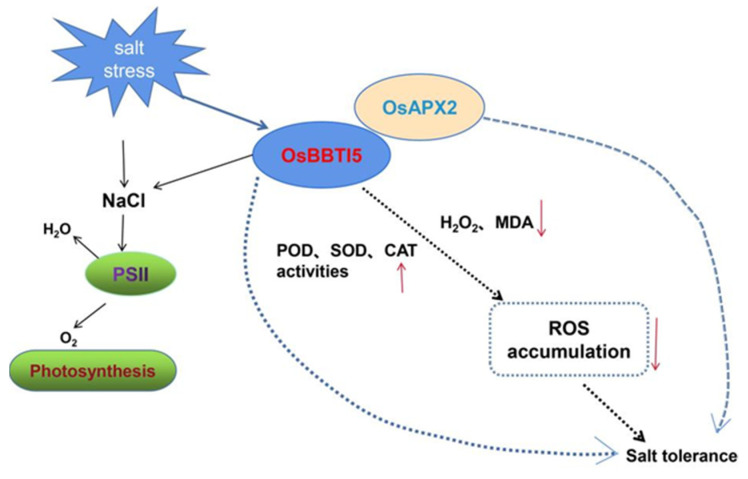
A proposed model illustrating how the *OsBBTI5*-RNAi, responsive to salt stress, enhances salt tolerance in rice.

Until now, the sole reported cloning of a Bowman–Birk-type protease inhibitor gene, *WRSI5*, in wheat was achieved using the 5′-race technique, and its overexpression in *Arabidopsis thaliana* demonstrated tolerance to 150 mM NaCl [45]. Recent research on BBI has primarily focused on crops like soybean and wheat, with limited relevance to stress responses in rice. However, our study provides novel insights by demonstrating that the *OsBBTI5* gene, belonging to the BBI family, is associated with the BR signaling pathway. Notably, RNA interference (RNAi) knockdown of this gene enhances salt tolerance in rice. We present, for the first time, a working model illustrating how salt-sensitive OsBBTI5-RNAi promotes salt tolerance (Figure 8). 

In the salt tolerance pathway, *OsBBTI5*-RNAi transgenic plants increased the expression of photosystem II (PSII) genes. Additionally, the *OsBBTI5* gene may interact with *OsAPX2* gene, leading to a reduction in the accumulation of ROS and thereby enhancing salt tolerance.

BR biosynthetic enzymes in rice have been studied mainly by phenotyping dwarf mutants, and the results indicate that BR is involved in many physiological processes in rice, including leaf elongation, tiller development, photogenesis, root differentiation, and reproductive growth [46]. BR signaling in *Arabidopsis* and rice (dicot and monocot models, respectively) is mediated by the receptor kinases *BRI1* and *OsBRI1* [47]. BR stimulates the activities of SOD, CAT, POD and APX, thereby reducing cold-induced damage [48]. Brassinolide (BL) improves plant tolerance to abiotic stress. In apples, exogenous BL increases the activities of SOD and CAT, thereby eliminating the salt stress-induced production of ROS [49]. BR immersion significantly increases SPAD, Pn and Tr, as well as Fm, Fv/Fm, and Fv/Fo in rice seedlings under NaCl stress, which protects the photosynthetic system of the plant and increases plant biomass [37]. In our study, the results from the BR assay revealed that rice with *OsBBTI5*-RNAi was less sensitive to exogenous brassinolide. Reducing the expression of *OsBBTI5* showed that a significant increase in POD, SOD, and CAT occurred after salt soaking, while a significant decrease in H_2_O_2_ and MDA occurred, which reduced the accumulation of ROS and improved the salt tolerance of transgenic plants in rice. In addition, the transcriptomic results indicated that the transgenic plants showed a significant increase in gene expression, mainly in photosynthesis-related and photosynthetic systems after salt immersion, which was an important factor contributing to the increase in salt stress.

Under normal conditions, exposure to NaCl leads to an increase in the activities of ascorbate peroxidase (APX) and glutathione reductase (GR) in rice roots. Simultaneously, the expression of *OsAPX* and *OsGR* is upregulated [50]. Rice has 8 *APX* genes that encode enzymes that function in the cytoplasm (*APX1* and *APX2*), peroxisomes (*APX3* and *APX4*), mitochondria (*APX5* and *APX6*), and chloroplasts (*APX7* and *APX8*) [51,52]. Overexpression of *OsAPX2* increases APX activity and improves stress tolerance. It is also shown that NaCl-induced expression of *OsAPx8* in rice roots requires Na(+) but not Cl(-) [53]. The yeast two-hybrid results in our study suggest that it is possible for *OsBBTI5* to act on stress tolerance through *OsAPX2* interactions.

Brassinosteroid (BR) and gibberellin (GA) are the two main hormones that regulate cell elongation in plants. Rice mutants that are insensitive to BR signaling typically exhibit stem and leaf elongation defects [54]. A member of the *GRAS* family, the *DLT* gene, is insensitive to BR expression, causing leaf bending, and affecting radicle elongation [55]. Coleoptile elongation and root inhibition assays show that rice overexpressing *OsPRA2* is less sensitive to exogenous brassinosteroid. BR regulates cell elongation by modulating GA metabolism in rice. For example, under physiological conditions, BR stimulates cell elongation by regulating the expression of GA metabolism genes, thereby promoting GA accumulation [56]. In addition, mutations in *LEA33* may affect grain size and seed germination in rice by reducing BR accumulation and promoting GA biosynthesis [57]. The overexpression of *OsOFP22* promotes SLR1 protein expression in response to GA-induced accumulation and represses the BR expression of signaling genes that ultimately regulate rice plant and grain size [58]. Similarly, in this study, the growth and development of transgenic plants after the downregulation of *OsBBTI5* expression were affected by high concentrations of GA_3_ more than BR. 

## 4. Materials and Methods

### 4.1. Plant Materials and Growth Conditions

Rice (*Oryza sativa* L. spp. Japonica) seedlings were grown in a greenhouse under standard rice growing conditions. Tobacco was grown in an artificial climate chamber at 26 °C under long-day conditions (16 h light/8 h darkness).

### 4.2. Vector Construction and Genetic Transformation in Rice

Two sets of specific primers, Os5F1/Os5R1 and Os5F2/Os5R2, were employed to amplify a 765 bp fragment corresponding to the full-length *OsBBTI5* cDNA. Subsequently, the PCR products were cloned into the pTCK303 vector using BamHI/KpnI and SpeI/SacI restriction sites through a two-step cloning process facilitated by a cloning kit (Vazyme, China, code: C113). The resulting plasmid, pTCK303-BBI5, was then introduced into *Agrobacterium tumefaciens* strain LBA4404. The transformation of transgenic rice plants was conducted following established procedures [59]. Briefly, mature seeds were sterilized and placed in an induction medium (NB, 2,4-D 2.5 mg/L, pH 5.8) for dark culturing at 28 °C. Following a two-week healing induction period, Agrobacterium and healing pellets were allowed to incubate in a dark culture at 28 ℃ for half an hour. Subsequently, the pellets were transferred to a co-culture medium (induction basal medium, AS 100 μM/L, pH 5.8) and underwent 2–3 washes with sterile water over a 3-day period. After the 3-day co-culture, the calli were washed 2–3 times with sterile water and then rinsed once with cephalexin 100 mg/L. These calli were then cultured on a screening medium containing induction medium, hygromycin 30 mg/L, and pH 5.8. Following 15 days of dark culture at 28 °C, the newly formed calli were subjected to an additional 3 days of dark culture at 28 °C. After a total of 15 days, the newborn calli were transferred to a differentiation medium (NB, 1.5 mg/L KT, 0.5 mg/L NAA, pH 5.8) and ultimately cultivated on a rooting medium (1/2 MS, sucrose 5%, Phytagel 2.5 g/L, pH 5.8) for a period ranging from 15 to 30 days. *OsBBTI5*-RNAi lines, exhibiting varying levels of expression, were generated and utilized for subsequent analyses.

### 4.3. Subcellular Localization

The full-length *OsBBTI5* cDNA without the termination codon and GFP cDNA were amplified using PCR primers Os5F3/Os5R3 and GFF/GFR (Table S4), and the resulting products were inserted into the *Bam*HI/*Hin*dIII-digested pCXSN (pCXSN-35SBBI5) using cloning kit (Vazyme, Nanjing, China, code: C113). Then, the plasmids of pCXSN35GFP (as positive control) and pCXSN35SBBI5 were, respectively, transformed into *Agrobacterium tumefaciens* strain GV3101. 

The single clone was picked and grown in an LB medium (containing rifampicin) on a shaker at 28 °C for 48 h and collected by centrifugation at 4000 rpm for 10 min. It was resuspended with MES resuspension (1 mL 500 mM MES + 1 mL 100 mM MgCl_2•_6H_2_O + 10 μL 500 mM AS, add ddH_2_O to 50 mL, pH 5.8) solution to OD_600_ = 1.0, left at room temperature for 2 h, and injected into tobacco leaves. Then the samples were observed after 2–3 days. The GFP fluorescence was monitored at 488 nm excitation using a laser confocal microscope (Leica SP8 STED, Germany).

### 4.4. Lamina Joint Assay

Leaf co-determination experiments were conducted by utilizing excised leaf segments, following previously established protocols [60]. Seeds were subjected to dark culture for 7 days in an incubator set at 30 °C, allowing the formation of two leaves. Subsequently, the entire segment, encompassing 1 cm of the second leaf, the leaf node, and 1 cm of the leaf sheath, was immersed in varying concentrations of 24-epibrassinolide for 48 h in the absence of light. The angles of lamina joint bending were measured using ImageJ software version 1.54a (http://rsbweb.nih. gov/ij/, accessed on 12 February 2023).

### 4.5. Salt Stress Treatment and Phenotypic Analysis of Transgenic Rice

Seeds were cultivated on a Murashige and Skoog (MS) solid medium containing 0, 40 mM, 50 mM, and 60 mM NaCl for a duration of 7 days. Two-week-old seedlings of both wild-type (WT) and *OsBBTI5*-RNAi varieties were cultured in hydroponic solution containing 40 mM, 80 mM, and 100 mM NaCl for 7 days. Additionally, rice seeds were placed on a 0.5× agar medium with varying concentrations of NaCl, 24-epibrassinolide (0.001 μM, 0.01 μM, 0.01 μM, 0.1 μM, 1 μM), or GA3 (0.1 μM, 1 μM, 10 μM, 100 μM) for 7 days, and seedling phenotypes were subsequently assessed. Each treatment was replicated three times, with 30 seedlings per replication.

### 4.6. RNA Extraction and Transcriptome Data Analysis

Rice seedlings treated with 40 mM NaCl for 3 days were stored into liquid nitrogen. RNA extraction was performed by using RNA-extraction kit (TransGen, Beijing, China, code:DP432). Three distinct samples, namely wild-type (WT), 40 mM NaCl-soaked, and *OsBBTI5*-RNAi samples, were subjected to differential expression analysis to elucidate specific responses.

The determination of differential gene expression involved the utilization of the Cuffdiff utility within the Cufflinks package. Transcripts exhibiting log2-fold changes of ≥1 (indicative of upregulated genes) and ≤(−1) (indicative of downregulated genes), with a *p*-value cut-off of ≤0.05, were considered significantly differentially expressed [61]. Subsequently, the identified DEGs underwent gene ontology (GO) enrichment analysis. Following the generation of DEGs for each region, as depicted in the Venn diagram analysis [62], the GO enrichment analysis was conducted to unravel the functional significance of the differentially expressed genes. 

### 4.7. Quantitative Real-Time PCR Analysis

qRT-PCR was performed using a Q1 real-time PCR system (Thermo Fisher Scineitific, Waltham, MA, USA). The system is a 10 µL volume: each reaction contains 5 μL of 2×Taq Pro Universal SYBR qPCR Master Mix (Vazyme, China), 0.4 μL of primers (10 μM), 1 μL of cDNA template, and added 10 μL of ddH2O. The procedure was performed according to the Q1 Semi-quantitative PCR Operation Manual. The rice actin gene was used as an internal reference. Quantitative PCR expression levels were calculated according to the 2^−ΔΔCT^ method. All experiments were performed with three biological replicates and three technical replicates.

### 4.8. Yeast Two-Hybrid Assay

The coding sequences of *OsBBTI5* and *OsAPX2* were, respectively, cloned into the pGBKT7 and pGADT7 vectors (Clontech, Mountain View, CA, USA), which were named pGBKT7-BBI5 and pGADT7-APX2. The resulting constructs and the corresponding empty vectors were then co-transformed into the yeast strain Golden Yeast in various combinations. The screening of the rice yeast library was conducted, resulting in the identification of 96 clones subjected to PCR validation. Upon reversing the validation outcomes, we proceeded to sequence a total of 210 candidate genes to explore potential interactions. Notably, this set included the *OsAPX2* gene. Interactions were detected on SD/-His-Leu-Trp and SD/-His-Leu-Trp+X-gal media. Transformation was performed according to the Yeast Two-Hybrid System User Manual (Clontech, Beijing China). All primers used in this assay are listed in Table S4.

## 5. Conclusions

This study employed a comprehensive approach to investigate the role of the *OsBBTI5* gene in rice under various conditions, shedding light on its potential functions and molecular interactions. The sequence analysis of *OsBBTI5* provided insights into its evolutionary relationships within the *BBI* gene family, its motif distribution, and the regulatory elements in its promoter region. Subcellular localization studies revealed the widespread distribution of *OsBBTI5* across organelles, suggesting its involvement in cellular processes. The generation of *OsBBTI5*-RNAi lines demonstrated the gene’s role in brassinosteroid (BR) signaling, with transgenic plants displaying insensitivity to BR and altered responses to 24-epibrassinolide and gibberellic acid (GA_3_). Furthermore, this study explored the impact of *OsBBTI5* on salt stress, revealing enhanced salt tolerance in *OsBBTI5*-RNAi transgenic rice plants. Physiological analyses, including measurements of peroxidase (POD) and superoxide dismutase (SOD) activities, hydrogen peroxide (H_2_O_2_) levels, and malondialdehyde (MDA) content, provided valuable insights into the mechanisms underlying salt stress responses. RNA sequencing and differential gene expression analysis unveiled distinct regulatory pathways, particularly in light-related processes, indicating the intricate role of *OsBBTI5* in the rice salt stress response. The identification of *OsAPX2* as an interacting partner of *OsBBTI5* through yeast two-hybrid assays suggests a potential molecular mechanism underlying salt tolerance in rice. These findings contribute to our understanding of the multifaceted functions of *OsBBTI5* and its implications in plant growth, development, and stress responses. Overall, this study provides a foundation for further research into the intricate molecular networks governing plant responses to environmental stimuli.

## Data Availability

Data are contained within the article and supplementary materials.

## Supplementary Materials

The following supporting information can be downloaded at: https://www.mdpi.com/article/10.3390/ijms25021284/s1.
