# Supplementary material for: RNAi-Mediated Suppression of OsBBTI5 Promotes Salt Stress Tolerance in Rice
Source: Int J Mol Sci. 2024 Jan 20;25(2):1284. doi: 10.3390/ijms25021284 (PMC10816146; doi:10.3390/ijms25021284)
Supplement: Supplementary file 1 [file ijms-25-01284-s001.zip › ijms-2699683-supplementary.pdf]

Supplemental Table S1. Summary of RNA-seq reads and assembly statics

Treat	Replicate	No. reads	Yield (Gb)	Q30 (%)	No. cleaned reads	No. reads uniquely aligned	%
Control	CK	47,186,596	7.08G	95.12	45,852,150	28,068,620	63.88
	CK	40,124,734	6.02G	92.31	38,802,000	23,513,714	62.69
	CK	44,142,562	6.62G	93.03	42,761,756	25,760,848	62.93
Salt	CK4	42,476,188	6.37G	92.4	41,398,322	37,359,978	93.37
	CK4	44,214,420	6.63G	93.53	43,079,210	39,219,949	94.45
	CK4	57,748,456	8.66G	93.99	55,871,914	50,960,554	94.55
Salt	KT39	44,726,454	6.71G	92.34	42,677,776	39,688,890	95.05
	KT39	43,198,124	6.48G	92.12	41,831,418	38,779,971	94.92
	KT39	43,719,896	6.56G	92.22	42,037,792	38,905,416	94.95

Supplemental Table S2. Enriched pathway by KEGG pathway analysis between salt-stressed *OsBBTI5*-RNAi and WT.

Term	Description	GeneRatio	BgRatio	p-value
dosa00940	Phenylpropanoid biosynthesis	120/1870	175/4991	2.06E-17
dosa00195	Photosynthesis	41/1870	48/4991	8.08E-12
dosa00904	Diterpenoid biosynthesis	36/1870	43/4991	5.14E-10
dosa00500	Starch and sucrose metabolism	100/1870	169/4991	4.88E-09
dosa00906	Carotenoid biosynthesis	25/1870	30/4991	3.11E-07
dosa00196	Photosynthesis - antenna proteins	15/1870	15/4991	3.89E-07
dosa00480	Glutathione metabolism	69/1870	122/4991	1.14E-05
dosa04626	Plant-pathogen interaction	118/1870	232/4991	1.44E-05
dosa04075	Plant hormone signal transduction	130/1870	263/4991	3.21E-05

Supplemental Table S3. Enriched pathway by KEGG pathway analysis between salt-stressed WT and WT.

Term	Description	GeneRatio	BgRatio	p-value
dosa00940	Phenylpropanoid biosynthesis	69/673	174/4877	5.58E-18
dosa00904	Diterpenoid biosynthesis	24/673	39/4877	5.09E-12
dosa00908	Zeatin biosynthesis	12/673	26/4877	6.54E-05
dosa00500	Starch and sucrose metabolism	39/673	163/4877	0.000283498
dosa00999	Biosynthesis of various plant secondary metabolites	19/673	65/4877	0.000861887
dosa00270	Cysteine and methionine metabolism	29/673	117/4877	0.000922152
dosa00400	Phenylalanine, tyrosine and tryptophan biosynthesis	16/673	51/4877	0.000941276
dosa04814	Motor proteins	23/673	86/4877	0.001019366
dosa00360	Phenylalanine metabolism	14/673	44/4877	0.00166508
dosa00900	Terpenoid backbone biosynthesis	16/673	56/4877	0.002804345

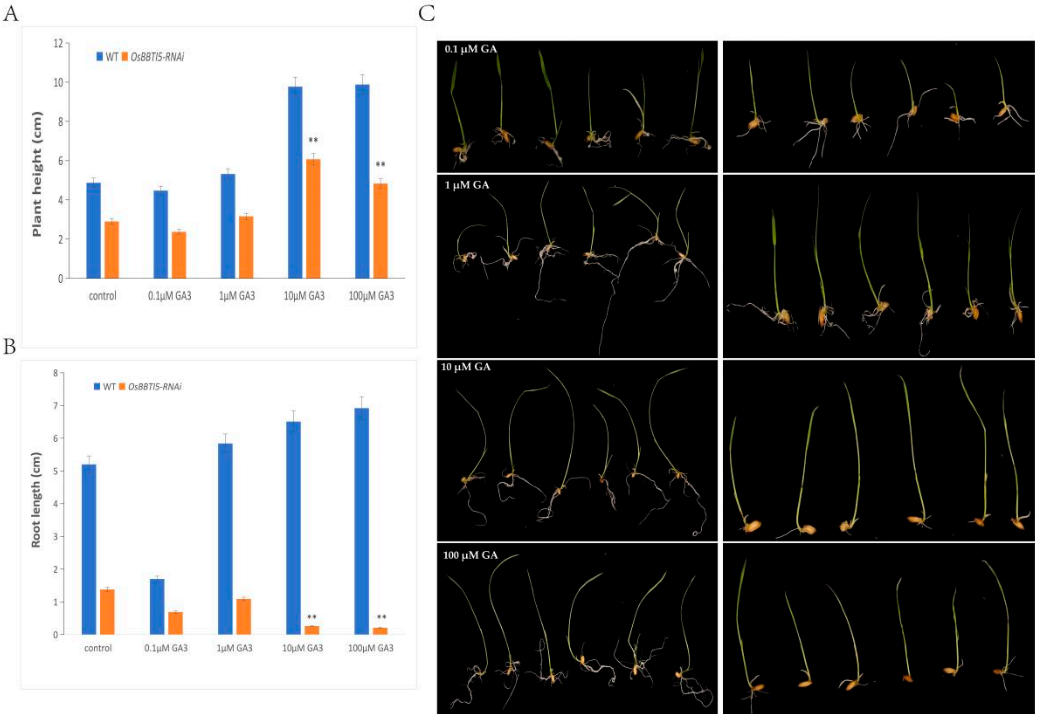

Figure S1. BR sensitivity assay test. (**A**,**B**) Measurements of plant heights and root lengths of WT and *OsBBTI5*-RNAi transgenic seedlings after 7 days of growing on MS medium containing diferent GA3 concentrations. Data are means ± SDs (n=30). (**C**) The growth of WT and *OsBBTI5*-RNAi plants grown on MS medium containing different GA3 concentration after 7 days. ** p < 0.01.

.

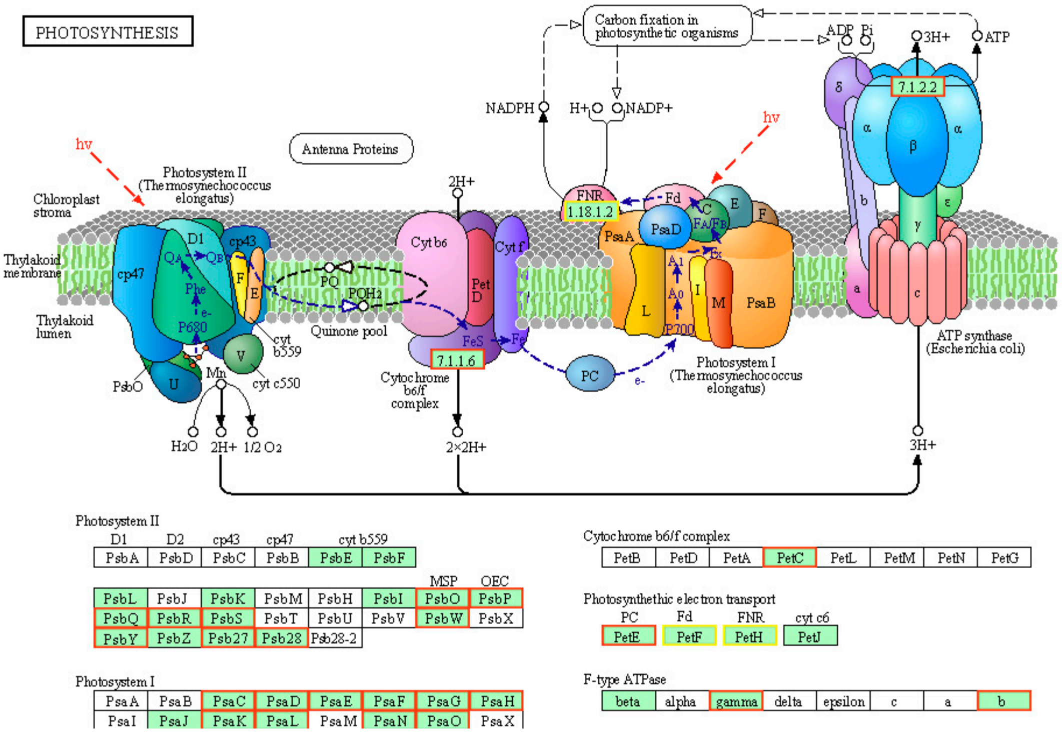

Figure S2. Differential genes of the photosynthetic pathway between *OsBBTI5*-RNAi and WT under 40 mM NaCl treatment.

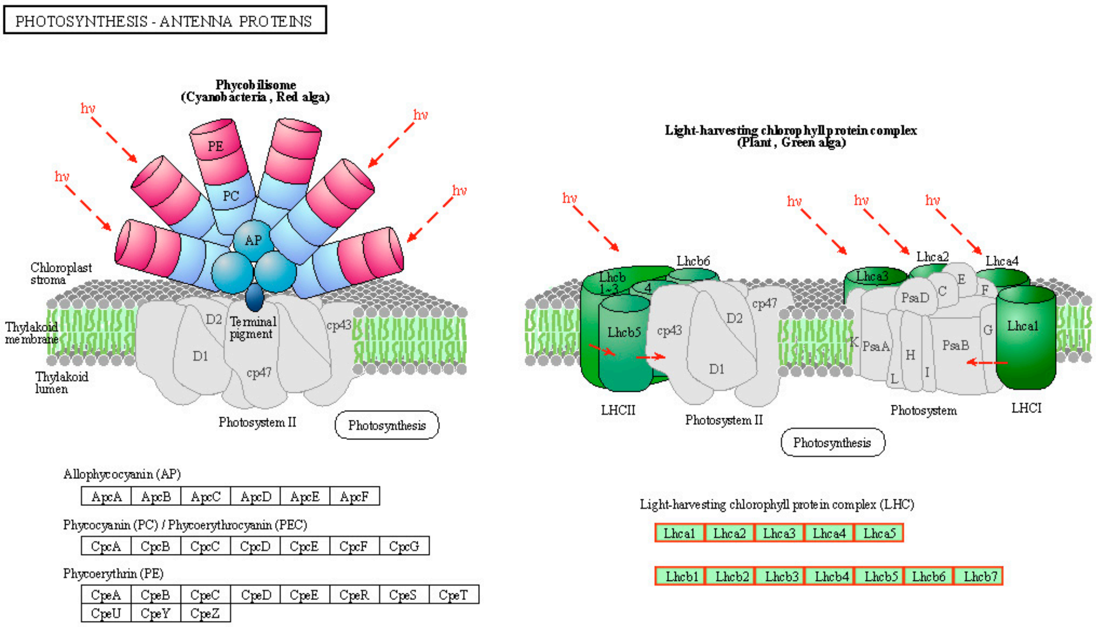

Figure S3. Differential genes of the photosynthesis-antenna proteins pathway between *OsBBTI5*-RNAi and WT under 40 mM NaCl treatment.

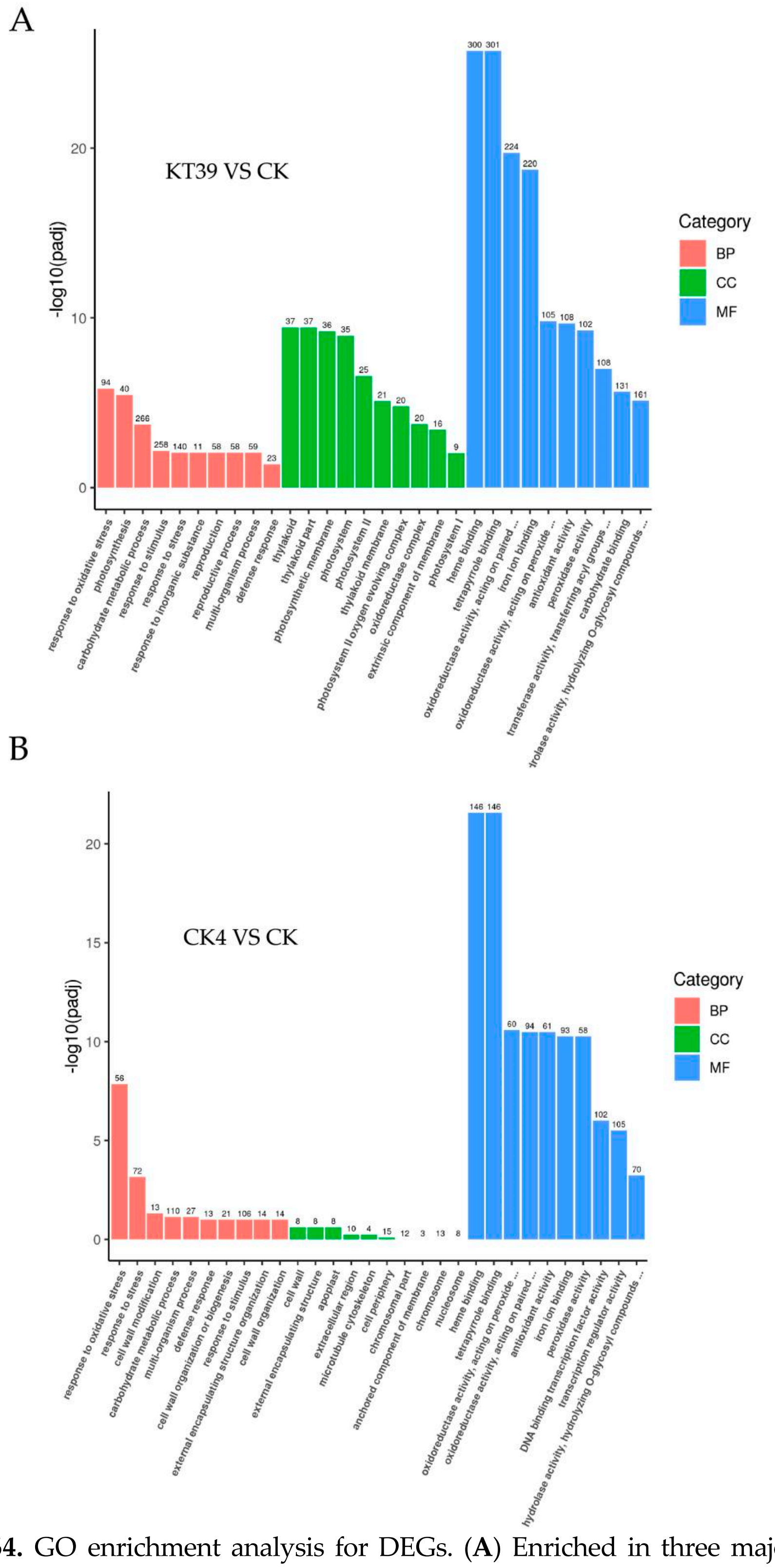

Figure S4. GO enrichment analysis for DEGs. (**A**) Enriched in three major classes of DEGs between KT39 and WT, including biological process (BP), cellular component (CC), and molecular function (MF). (**B**) Enriched in three major classes of DEGs between CK4 and WT, including biological process (BP), cellular component (CC), and molecular function (MF). KT39 is a synonym with the *OsBBTI5*-RNAi lines under 40 mM NaCl treatment; CK4 is a synonym with the the wild-type lines under 40 mM NaCl treatment; CK is a synonym with the wild-type lines under normal nutrient solution.

Table S4. List of gene-specific primers used for gene cloning.

	Gene and primer name	Forward primer (5' to 3')	Reverse primer (5' to 3')
**Gene cloning for pTCK303-BBI5**	*OsBBTI5* (Os5F1/Os5R1)	gacctcgagggtaccATGAGCAACACTACCATGGCT	gactctagaggatccCTAGTTCTCCGCTCGGGGTTT
**Gene cloning for pTCK303-BBI5**	*OsBBTI5* (Os5F2/Os5R2)	gggaaattcgagctcCTAGTTCTCCGCTCGGGGTTT	tcaatcgatactagtATGAGCAACACTACCATGGCT
**Gene cloning for pCXSN-35SBBI5**	*OsBBTI5* (Os5F3/Os5R3)	GTTCTCCGCTCGGGGTTTGCAC	gccagtgccaagcttATGAGCAACACTACCATGGCT
**Gene cloning for pCXSN-35SBBI5**	*GFP* (GFF/GFR)	ttcgctagtggatccTTACTTGTACAGCTCGTCCAT	ATGGTGAGCAAGGGCGAGGTTCTCCGCTCGGGGTTTGCA
**Gene cloning for pGBKT7-BBI5**	*OsBBTI5* (Osp5F1/Osp5R1)	ATGGCCATGGAGGCCGAATTCATGAGCAACACTACCATGGCTAC	CCGCTGCAGGTCGACGGATCCCTAGTTCTCCGCTCGGGGTTTGC
**Gene cloning for pGADT7-APX2**	*OsAPX2* (OsApF/OsApR)	GCCATGGAGGCCAGTGAATTCATGGGCAGCAAGTCGTACC	AGCTCGAGCTCGATGGATCCTTATTCCTCAGCAAATCCCA
**For QRT-PCR**			
	OsBBTI5 (RTB5F/RTB5R)	GGAAGCTCATCTGCGAGGACATCT	AGGTCGGCGGGTTCATCTTGTTG
	DLT (DLTF/DLTR)	GCAAGTCCTCCATGTTAGTGGTCCT	GCAATCTTCTCAATGTCGCCAAGGT
	OsBR6ox (OsBR6oxF/OsBR6oxR)	CAGGTACGGGAGCGTGTT	TGAAGCCTTGGTAGTAGTTGGT
	OsBZR1 (OsBZR1F/OsBZR1R)	TGCCATCGCCGCCAAGATCTTCA	AACGGGTCGACGTCCCAGTCCGG
	D61 (D61F/D61R)	GAGGAGCGGTTGTTGGTGTATGATT	TCTCGGTGAATGATGTGAGGAATGC
	OsSPY (OsSPYF/OsSPYR)	CTGGCTCAGTCCATGCTCATAATGT	CTCGGAGGCTCATTCTCAGTTCTTG
	D11 (D11F/D11R)	GCAAGTCCTCCATGTTAGTGGTCCT	GCAATCTTCTCAATGTCGCCAAGGT
	Actin (ActinF/ActinR)	GACCCAGATCATGTTTGAGACC	CATCACCAGAGTCCAACACAATAC
